# How equitable is health spending on curative services and institutional delivery in Malawi? Evidence from a quasi-longitudinal benefit incidence analysis

**DOI:** 10.1186/s12939-022-01624-5

**Published:** 2022-02-18

**Authors:** Martin Rudasingwa, Edmund Yeboah, Valéry Ridde, Emmanuel Bonnet, Manuela De Allegri, Adamson Sinjani Muula

**Affiliations:** 1 Heidelberg Institute of Global Health, University Hospital & Medical Faculty, Heidelberg University, Heidelberg, Germany; 2 CEPED, Institute for Research on Sustainable Development, IRD-Université de Paris, ERL INSERM SAGESUD, Paris, France; 3 IRD, UMR 215 Prodig, CNRS, Université Paris 1 Panthéon-Sorbonne, AgroParisTech, 5, Cours des Humanités, F-93 322 Aubervilliers, Cedex France; 4 School of Public Health and Family Medicine, College of Medicine, University of Malawi, Blantyre, Malawi; 5 Kamuzu University of Health Sciences, Blantyre, Malawi

**Keywords:** Benefit incidence analysis, health spending, inequality, health care utilization, Malawi

## Abstract

**Background:**

Malawi is one of a handful of countries that had resisted the implementation of user fees, showing a commitment to providing free healthcare to its population even before the concept of Universal Health Coverage (UHC) acquired global popularity. Several evaluations have investigated the effects of key policies, such as the essential health package or performance-based financing, in sustaining and expanding access to quality health services in the country. Understanding the distributional impact of health spending over time due to these policies has received limited attention. Our study fills this knowledge gap by assessing the distributional incidence of public and overall health spending between 2004 and 2016.

**Methods:**

We relied on a Benefit Incidence Analysis (BIA) to measure the socioeconomic inequality of public and overall health spending on curative services and institutional delivery across different health facility typologies. We used data from household surveys and National Health Accounts. We used a concentration index (CI) to determine the health benefits accrued by each socioeconomic group.

**Results:**

Socioeconomic inequality in both public and overall health spending substantially decreased over time, with higher inequality observed in overall spending, non-public health facilities, curative health services, and at higher levels of care. Between 2004 and 2016, the inequality in public spending on curative services decreased from a CI of 0.037 (SE 0.013) to a CI of 0.004 (SE 0.011). Whiles, it decreased from a CI of 0.084 (SE 0.014) to a CI of 0.068 (SE 0.015) for overall spending in the same period. For institutional delivery, inequality in public and overall spending decreased between 2004 and 2016 from a CI of 0.032 (SE 0.028) to a CI of -0.057 (SE 0.014) and from a CI of 0.036 (SE 0.022) to a CI of 0.028 (SE 0.018), respectively.

**Conclusions:**

Through its free healthcare policy, Malawi has reduced socioeconomic inequality in health spending over time, but some challenges still need to be addressed to achieve a truly egalitarian health system. Our findings indicate a need to increase public funding for the health sector to ensure access to care and financial protection.

**Supplementary Information:**

The online version contains supplementary material available at 10.1186/s12939-022-01624-5.

## Background

Ensuring equitable access to health services across all socioeconomic groups is a global challenge related to achieving Universal Health Coverage (UHC). Including UHC in the Sustainable Development Goal 3 indicates a global aspiration to ensure equitable access to quality care and health financial protection for all [1]. While UHC ranks high on the global health agenda, low-and middle-income countries, especially in sub-Saharan Africa (SSA), still face high health inequalities [2, 3]. Being aware of these inequalities and the urgent need to overcome them, SSA countries and their development partners are progressively increasing investments and efforts to build and sustain more inclusive health systems.

Reforms aimed at UHC, including user fee removal policies, targeted subsidies, and performance-based financing, have been implemented across SSA with the explicit aim of reducing existing inequalities in access [4–6]. While evidence on the equity impact of these reforms is increasing, [7–9] limited information is available on whether and how implementing these reforms has altered the distributional incidence of health spending.

Following the structural adjustment programs and the Bamako initiatives in the 1980s, many African countries were pushed to introduce user fees for health services, which led to the underuse of health services, especially for vulnerable groups [10, 11]. In contrast with many sub-Saharan countries, Malawi resisted the introduction of user fees and has continued on its traditional path of free care at the point of use at public health facilities [12]. Beyond its free healthcare policy, to redress pronounced health inequities observed in Malawi's healthcare delivery system in the 1990s and early 2000s [13], the government implemented additional reforms to increase coverage of curative services and institutional delivery. From 2004, the Malawi government centered healthcare delivery around providing an Essential Healthcare Package (EHP) to guide, more specifically, both the planning and funding of healthcare provision. The health services targeted in the EHP include care for infectious and non-communicable diseases, reproductive health, and child health, with services intended to be provided free of charge at the point of use in public facilities. Additionally, since 2006, selected services targeting primarily maternal and neonatal health are also available free of charge in private-not-for-profit religious facilities (CHAM) contracted by the Ministry of Health through Service Level Agreements (SLA) [14].

Though the per capita health spending from all sources of funds has increased over time, the Government of Malawi faces financial constraints in financing the EHP services. In 2011, the domestic budget allocated to health was estimated at 7.2% [15], less than half of the 15% pledged by the heads of states of the African Union countries in the 2001 Abuja Declaration [16]. In the same year, public spending on health accounted for 19% of total funds, while external contributions and OOPE accounted for 74% and 7%, respectively [17]. The proportion of public health expenditure to GDP was estimated at 2.5% in the fiscal year 2017/2018 [18], half of the recommended share of GDP that low-and-middle-income countries have to spend on health to achieve substantial progress in UHC [19]. For the fiscal year 2013/2014, the public financial gap to finance the EHP was estimated at USD 358 million [20]. As a consequence of the underfunding of the EHP policy, patients still incur substantial out-of-pocket payments for the services included in the EHP, hindering adequate access to care [21–25]. In particular, the underfunding of the EHP services under SLA contracts pushed some CHAM facilities to reintroduce user fees for curative services [26]. Previous studies revealed that the more affluent disproportionately use both curative services [27] and maternal services [28] and hence incur higher OOPE than the poorer segments of the population [27]. A recent study by Mchenga and colleagues indicated that OOPE caused 9.37% of households to face catastrophic health expenditure, leading to an increase of the poverty gap of almost 2.54% [29]. Though the literature indicates that the community is well aware of its health coverage rights, financial shortcomings in implementing EHP policy hamper its effectiveness [22]. Arnold and colleagues [30] have suggested that the EHP framework redesign by considering health equity gaps could lead to more equitable use of health services and reduced direct payments.

In addition to reforming the EHP, the government of Malawi has piloted results-based financing in several districts to test how the introduction of purchasing reforms could sustain other ongoing efforts and advance progress towards UHC. The Results-Based Financing for Maternal and Newborn Health (RBF4MNH) initiative was piloted in four districts (Balaka, Dedza, Mchinji, Ntcheu) between 2013 and 2018 and explicitly targeted obstetric care services through a combination of supply and demand-side incentives. The Support for Service Delivery Integration Performance-Based Incentive (SSDI-PBI) program was implemented between 2015 and 2017 in three districts (Chitipa, Nkhotakota and Mangochi). It targeted a broader range of maternal, reproductive, and child services by implementing supply-side incentives. Existing evidence suggests that the RBF4MNH initiative led to improvements in the quality of obstetric care services but yielded limited effect on the use of those services [31]. The SSDI-PBI scheme has been shown to produce remarkable changes in providing essential health services, but with a high heterogeneity across health services and health facilities [32].

A few studies have looked at the changes in health financing flows and health spending distribution across districts. A study by Borghi and colleagues explored the process of receiving and allocating funding at the district level and found that all funding sources were disproportionately allocated to wealthier districts, with OOPE being the most inequitably distributed, followed by public spending and external aid. In addition, this study revealed that the underfunding of health services at the district level and high OOPE were associated with high neonatal mortality rates [17]. Mann and colleagues [33] indicated that the increase of health financing on maternal and childcare led to reduced maternal and under-5 child mortality rates. These authors, however, reported that Malawi's high dependence on external resourcing presents a problem with the financial sustainability of healthcare. These studies, however, fall short of developing comprehensive analyses of the equity implications of Malawi's health financing policies, looking specifically at the distribution of health spending at the population level. Moreover, these studies do not explore changes over time in relation to the policy reforms rolled out in the country.

**Fig. 1:**
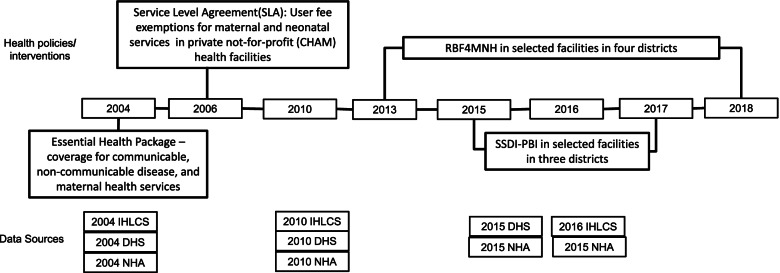
Timeline of health policies and interventions targeting curative and maternal services in Malawi

Our study aims to fill this knowledge gap by estimating the distributional incidence of both public and overall health spending (including donor and private health expenditure) on curative services and institutional delivery (childbirth at a health facility) at three different time points in Malawi. Our ambition was to explore how and to what extent equality in health spending evolved over time , also as a function of UHC reforms being implemented in Malawi (Fig. 1).

## Methods

### Study design

We applied a Benefit Incidence Analysis (BIA) to assess the distributional incidence of both public and overall health spending on curative services and institutional delivery at three time points. Public health spending refers to public subsidies allocated to health facilities for the provisions of care. Overall spending refers to all sources of health spending allocated to health facilities in terms of public spending, external support and out-of-pocket expenditure (OOPE). BIA measures whether the financial benefits of health services reach individuals across socioeconomic groups equally at a specific point in time [34, 35]. BIA has traditionally been employed to assess the distributional incidence of public spending. Given the growing global emphasis on fostering UHC by combining multiple financing mechanisms, McIntyre and Ataguba recently argues in favor of expanding the scope of the BIA methodological approach to assess the distribution incidence of overall health system spending. Our work builds on their theoretical postulations and their specific methodological guidance [34].

Due to the nature of BIA methodology and the available data, it was impossible to conduct strictly-speaking longitudinal analysis. Therefore, we describe our study as quasi-longitudinal. The computation of BIA relies on two datasets: data on health service utilization stratified by socio-economic status and data on the unit costs of different types of health services. In other words, BIA expresses in monetary terms the distribution of health benefits. As such, BIA aims to capture the extent to which investments in the health sector reach equally all strata of the population. To perform this analysis, we used data from available nationally representative repeated cross-sectional household surveys and National Health Accounts (NHA) for health service utilization and health spending, respectively. Before deciding on the time points of our analysis, we depicted and attempted to match, to the extent possible, for three time points: (1) available health policies and interventions (Fig. 1) that were implemented in Malawi to foster progress towards universal coverage of curative and maternal services; (2) the household survey data on utilization of curative services and institutional delivery; and (3) available data on health spending on curative services and institutional delivery available. We repeated the BIA at the selected three time points to explore changes in the distributional incidence of health spending in relation to the different UHC reforms implemented in the country.

Table S1 in the Additional file 1 illustrates which variables were used from each household survey, for each year, and briefly describes the sampling strategy of each survey. The repositories of the used datasets are provided under availability of data and materials.

**Table 1:** Variables and data sources

Variables and data sources	Health care providers	Data sources (years)	NHA data (year)	Additional data sources for seasonality adjustment (year)	Sources for OOP unit cost adjustment
Curative health service utilization by adults and children in the prior two weeks	Public health facilities, mission health facilities, and private health facilities	IHLCS (2004;2010;2016)	2004 2010 2015	HMIS (2014-2018)	Nakovics et al. 2020 [27]
Annual institutional deliveries	Public hospitals, public health centers, mission hospitals, mission health centers, and private facilities	DHS (2004;2010;2015)	2004 2010 2015	HMIS (2014-2018)	Chinkhumba et al. 2017 [37]

### Data sources and variable measurement

#### Health care utilization

We derived our data on health care utilization from the Integrated Household Living Condition surveys (IHLCS) for curative services and the Demographic and Health Surveys (DHS) for institutional delivery. These nationally representative household surveys, normally conducted every five years, contain data on the utilization of curative services and institutional deliveries differentiated by provider typology and a measure of socioeconomic status (SES), allowing us to categorize individuals by weighted SES quintiles. Table 1 indicates the health variables we extracted from each household survey.

As a ranking variable to build socioeconomic strata, we used per capita consumption expenditure based on the total household food and non-food expenditure for IHLCS data sets and the household-wealth-index factor scores generated through the principal components analysis based on household material asset ownership for DHS data sets.

We estimated the annual visits to curative services and institutional deliveries in the study year for individuals across different socioeconomic groups. We used a binary variable for curative services indicating whether the respondent had used curative services in the previous 14 days and a binary variable for institutional delivery indicating whether a woman had delivered in a facility in the prior twelve months. Counts of curatives services were annualized to obtain yearly counts by multiplying the visits recorded for the 14-day recall period by 26. We categorized curative services and institutional delivery by different health facilities types depending on data availability in each survey and NHA.

### Seasonality adjustment

Due to seasonal variation of disease incidences and use of health services, the literature indicates that the annualized utilization of health services may be underestimated or overestimated based on the period of data collection [36]. To account for these seasonal variations, we conducted a sensitivity analysis by adjusting the utilization of curative services and institutional delivery from the household surveys by the monthly seasonal variations in the use of these services. We built a monthly seasonality index using data from the 2014-2018 Health Management Information System (HMIS). We estimated averages of monthly health care utilization reported in the HMIS between 2014 and 2018 and used these averages to calculate the monthly seasonality indices. We then accounted for seasonal variations in the utilization of curative services and institutional delivery using corresponding seasonality indices depending on the months for which health service utilization was reported in the household surveys.

### Unit cost

We derived data on health spending from the National Health Accounts (see Fig. 1 and table 1). We estimated the unit cost using recurrent public spending, donor spending and household OOPE from the National Health Accounts. We applied the constant unit subsidy assumption for the public and donor spending to estimate the unit subsidy at different types of health facilities. We determined the unity subsidy of each type of health service at each type of health facility by dividing the total health spending for one type of service by the total utilization of that service at this health facility. For OOPE, we relied on the constant unit cost assumption for each quintile based on the percentage of OOPE incurred by each quintile at different types of health facilities. We adjusted the OOPE across quintiles based on the works by Nakovics et al. [27] for curative services and by Chinkhumba et al. [37] for institutional delivery. The OOPE adjustment was based on the fact that the individuals belonging to different SES quintiles generally display different OOPE at different types of facilities. Hence, using a constant unit OOPE at each type of facility would overestimate the OOPE incurred by the bottom SES quintiles. The studies by Nakovics et al. [27] and Chinkhumba et al. [37] indicated that the least poor incurred approximately twice as much OOPE for curative services and one third more OOPE for institutional delivery than the poorest segment of the population. We estimated the unit cost for each quintile and each type of health service at each health facility by dividing the total OOPE incurred by that quintile for that service at the same health facility by the total utilization accrued to that quintile for that service at that health facility.

### Analytical approach

We computed traditional BIA by measuring only the distributional incidence of public spending and comprehensive BIA by looking at the distributional incidence of overall health spending, including public and donor subsidy allocation to facilities as well as OOPE incurred by individuals. Our choice was motivated by a wish to provide a comprehensive health system assessment, in line with the policy intention of the reforms implemented to promote and sustain increased service coverage in the country. Based on the data availability (table 1), we decomposed our analysis by different health facility typologies for both curative services (public facilities vs faith-based facilities vs private facilities) and institutional delivery (public health centers vs public hospitals vs faith-based health centers vs faith-based hospitals vs private facilities) and each year. To determine the total financial health benefits at each type of health facility, we multiplied the unit subsidy or unit cost by the total utilization of health services at each type of health facility.

We used concentration indices to measure the degree of inequality in the distribution of public and overall health spending on curative services and institutional delivery across socioeconomic groups. The concentration index (CI) quantifies the degree of wealth-related inequality and ranges from -1.0 to +1.0. The CI takes a negative (positive) value when the financial health benefit is concentrated disproportionately among the poor (least-poor). If the CI is close to zero, a lower degree of inequality is present, and if it is zero, there is an absence of wealth-related inequality [35]. We adjusted the concentration indices by the sampling weights of the IHLCS and DHS household surveys to scale up sample-specific estimates to reflect the national population.

The standardized concentration index (*C*_*h*_) is estimated as follows [35]:

$${C}_h=\frac{2 Cov\ \left({h}_i,{R}_i\right)}{\mu }$$

Where *h*_*i*_ is the health variable (e.g. health care utilization) for individual ί, μ is the mean of health variable, *R*_*i*_ is individual i's fraction socioeconomic rank, and *Cov* (*h*_*i*_, *R*_*i*_) is the covariance.

We used convenient regression [38] to calculate the standard errors of the concentration index. The formula is:

$$2\sigma {\displaystyle \begin{array}{c}2\\ {}R\end{array}}\left[\frac{h_i}{\mu}\right]=\alpha +{\beta R}_i+{\varepsilon}_i$$

Where $$2\sigma {\displaystyle \begin{array}{c}2\\ {}R\end{array}}$$ is the variance of the fractional rank variable, *β* is the estimator of the concentration index.

We performed a dominance test at a significance level of 5%. The dominance test is used to statistically verify if a determined pro-poor or pro-least-poor distribution holds across the entire distribution of the socioeconomic variables [34, 35], especially when it is not clear if a distribution is pro-poor or pro-least-poor [39].

## Results

Descriptive statistics of health service utilization by individuals belonging to different socioeconomic quintiles and the unity subsidies/costs of health services at health facility typologies included in this study are reported in the Additional file 1.

### Benefit incidence of public spending on curative services

Table 2 reports the distributional incidence of public health spending on curative services at public and CHAM health facilities. With a concentration index of 0.037 (*p* < 0.01), total public spending was slightly pro-least-poor in 2004 but shifted to steady equality in 2010 and 2016. By breaking down public spending at types of health facilities, public health facilities were approximately equally distributed at all years while CHAM health facilities disproportionately benefited the least poor over time with a concentration index of 0.180 (*p* < 0.010) in 2004, 0.190 (*p* < 0.05) in 2010 and 0.187 (*p* < 0.01) in 2016. The overall inequality and the inequality at public health facilities slightly declined over time. However, the inequality at CHAM health facilities persisted over time with insignificant variation in the range of 1 to 5.5%.

**Table 2:** Distribution of financial health benefits of public spending on curative services

Health care provider	2004	2010	2016	Diff 2010-2004	Diff 2016-2010	Diff 2016-2004
	CI (SE)	CI (SE)	CI (SE)	CI (SE)	CI (SE)	CI (SE)
All public and CHAM health facilities	0.037^a^*** (0.013)	0.028^b^ (0.021)	0.004^c^ (0.011)	-0.009 (0.025)	-0.024 (0.024)	-0.033** (0.017)
Public health facilities	0.022^c^ (0.013)	0.014^a^ (0.023)	-0.006^a^ (0.011)	-0.008 (0.026)	-0.020 (0.025)	-0.028 (0.017)
CHAM health facilities	0.180^a^*** (0.038)	0.190^a^** (0.089)	0.187^a^*** (0.044)	0.010 (0.097)	-0.003 (0.099)	0.007 (0.058)

Notes: *CI *concentration index, *SE *standard errors; dominance test: a = dominance, b= non-dominance, c= curves cross;

*, **, *** statistically significant at the 10%, 5%, and 1% levels, respectively

### Benefit incidence of overall spending on curative services

Table 3 reports the distributional incidence of overall spending on curative services. A general picture indicates that overall health spending on curative services disproportionately benefited the least poor except for the public health facilities in 2016, where it was evenly distributed. The overall inequality of health spending on curative services in favor of the least poor increased by 36% between 2004 and 2010 from a CI of 0.084 (*p* < 0.01) to a CI of 0.114 (*p* < 0.01) but decreased by 40% to a CI of 0.068 (*p* < 0.01) in 2016. When we break down the overall spending on curative services at health facility typologies, the inequality significantly decreased between 2010 and 2016 at public health facilities. The inequality at public health facilities increased by 74% between 2004 and 2010 from a CI of 0.047 (*p* < 0.01) to a CI of 0.082 (*p* < 0.01), but considerably decreased by 91% towards equality in 2016 (CI = 0.007, not dominant). A similar distribution pattern of overall spending is observed at CHAM health facilities but with slight changes between years. The inequality at CHAM health facilities increased by 15% between 2004 and 2010 from a CI of 0.209 (*p* < 0.01) to a CI of 0.241 (*p* < 0.01), but decreased by 19% to a CI of 0.196 (*p* < 0.01) in 2016. A different picture is observed at private health services where the inequality of overall spending in favor of the least poor remained steady over time with a CI of 0.275 (*p* < 0.01) in 2004, a CI of 0.266 (*p* < 0.01) in 2010 and a CI of 0.282 (*p* < 0.01) in 2016.

**Table 3:** Distribution of financial health benefits of overall spending on curative services

Health care provider	2004	2010	2016	Diff 2010-2004	Diff 2016-2010	Diff 2016-2004
	CI (SE)	CI (SE)	CI (SE)	CI (SE)	CI (SE)	CI (SE)
All health facilities	0.084^a^*** (0.014)	0.114^a^*** (0.021)	0.068^a^*** (0.015)	0.03 (0.025)	-0.046* (0.026)	-0.016 (0.021)
Public health facilities	0.047^a^*** (0.013)	0.082^a^*** (0.023)	0.007^c^ (0.011)	0.035 (0.027)	-0.075*** (0.026)	-0.040* (0.018)
CHAM health facilities	0.209^a^*** (0.04)	0.241^a^** (0.093)	0.196^a^*** (0.045)	0.032 (0.102)	-0.045 (0.103)	-0.013 (0.062)
Private health facilities^+^	0.270^a^** (0.125)	0.266^a^*** (0.083)	0.282^a^*** 0.034	-0.004 (0.150)	0.016 (0.090)	0.012 (0.130)

Note : *CI *concentration index, *SE *standard errors; dominance test: a = dominance, b= non-dominance, c= concentration curve and line of equality cross; +: for private health facilities, only the OOP expenditure was included

*, **, *** statistically significant at the 10%, 5%, and 1% levels, respectively

Table 4 shows how the magnitude of the inequality in public and overall spending on curative services evolved across health provider typology. In general, the distribution of health spending did not change much over time; it mostly remained constant except for total public spending and overall spending at public health facilities that shifted from a low pro-least poor inequality to equality. The inequality was low at public facilities and moderate at CHAM and private facilities for both public and overall health spending.

**Table 4:** Changes of the inequality magnitude of public and overall spending on curative services across health care provider typology

Health spending	Year	All health facilities	Public health facilities	CHAM health facilities	Private health facilities
Public spending	2004	Low least poor	Equal	Moderate least poor	n/a
	2010	Equal	Equal	Moderate least poor	n/a
	2016	Equal	Equal	Moderate least poor	n/a
Overall spending	2004	Low least poor	Low least poor	Moderate least poor	Moderate least poor
	2010	Low least poor	Low least poor	Moderate least poor	Moderate least poor
	2016	Low least poor	Equal	Moderate least poor	Moderate least poor

Notes: Non-Significant CIs were considered equal, 1- -0.346: High pro-poor, -0.345 - 0.150: Moderate pro-poor, -0.149-0: Low pro-poor, 0.346-1: High least poor, 0.150-0.345: Moderate least poor, 0-0.149: Low least poor. na: not applicable

### Benefit incidence of public spending on institutional delivery

Table 5 shows the distributional incidence of public spending on institutional delivery at public health facilities (health centers and hospitals). Total public spending at public health facilities tended towards equality in 2004 and 2010 but shifted to a slight pro-poor benefit in 2015 with a concentration index of -0.057 (*p* < 0.01). Public hospitals and health centers were pro-least poor and pro-poor, respectively, between 2004 and 2015. The pro-least-poor inequality at public hospitals declined continually over time with an inequality reduction of 13% and 50% between 2004-2010 and 2010-2015, respectively. A different picture is observed at public health centers where the pro-poor inequality increased over time with an inequality increase of 17% and 97% between 2004-2010 and 2010-2015, respectively. In approximately ten years, the pro-least-poor inequality at public hospitals declined by 56%, while the pro-poor inequality at public health centers increased by 140%.

**Table 5:** Distribution of financial health benefits of public spending on institutional delivery

Health care provider	2004	2010	2015	Diff 2010-2004	Diff 2015-2010	Diff 2015-2004
	CI (SE)	CI (SE)	CI (SE)	CI (SE)	CI (SE)	CI (SE)
Public health facilities	0.032b (0.028)	0.001b (0.017)	-0.057a*** (0.014)	-0.031 (0.029)	-0.058*** (0.022)	-0.089*** (0.028)
Public hospitals	0.145a*** (0.047)	0.126a*** (0.025)	0.063a*** (0.024)	-0.019 (0.049)	-0.063 (0.035)	-0.082 (0.049)
Public health centers	-0.065a* (0.027)	-0.078a** (0.024)	-0.154a*** (0.018)	-0.013 (0.049	-0.076** (0.030)	-0.089*** (0.032)

Note: *CI *concentration index, *SE *standard errors; dominance test: a = dominance, b= non-dominance, c= concentration curve and line of equality cross

*, **, *** statistically significant at the 10, 5, and 1% levels, respectively

### Benefit incidence of overall spending on institutional delivery

Table 6 illustrates the distributional incidence of overall spending on institutional delivery. Generally, overall spending at public and CHAM hospitals benefited the least poor women, while the overall spending at public health centers displayed a pro-poor distribution. Overall spending at CHAM health centers and private health facilities equally favored all women. The total health spending at all health facilities was evenly distributed in 2004 and 2015 but was pro-least poor in 2010 with a concentration index of 0.078 (*p* < 0.01). Public hospitals benefited disproportionately the least poor for all years. However, this inequality declined continually over time, by 9% between 2004 and 2010 from a CI of 0.135 (*p* < 0.01) to a CI of 0.123 (*p* < 0.01) and by 40% between 2010 and 2015 to a slight pro-least poor inequality with a CI of 0.074 (*p* < 0.01). CHAM hospitals were pro-least poor in 2004 and 2010 but declined towards equality in 2015. The inequality at CHAM hospitals slightly declined by 14% between 2004 and 2010 from a CI of 0.154 (*p* < 0.01) to a CI of 0.132 (*p* < 0.01) and considerably declined by 82% to equality between 2010 and 2015. The pro-poor inequality at public health centers moderately declined by 27% between 2004 and 2010 from a CI of -0.106 (*p* < 0.01) to a CI of -0.077 (*p* < 0.01) but considerably increased by 88% to a CI of -0.145 (*p* < 0.01) in 2015.

**Table 6:** Distribution of financial health benefits of overall spending on institutional delivery

Health care provider	2004	2010	2015	Diff. 2010–2004	Diff. 2015–2010	Diff. 2015–2004
	CI (SE)	CI (SE)	CI (SE)	CI (SE)	CI (SE)	CI (SE)
All health facilities	0.036b (0.022)	0.078a*** (0.021)	0.028b (0.018)	0.042 (0.030)	-0.05* (0.027)	-0.008 (0.028)
Public health facilities	0.033a (0.024)	0.006b (0.017)	-0.071a*** (0.014)	-0.027 (0.029)	-0.077*** (0.022)	-0.104*** (0.028)
Public hospitals	0.135a*** (0.041)	0.123a*** (0.025)	0.074a*** (0.025)	-0.012 (0.048)	-0.049 (0.035)	-0.061 (0.048)
Public health centers	-0.106a*** (0.027)	-0.077a*** (0.024)	-0.145a*** (0.018)	0.029 (0.036)	-0.068** (0.030)	-0.039 (0.032)
CHAM health facilities	0.121a*** (0.042)	0.056a (0.041)	-0.037b (0.044)	-0.065 (0.059)	-0.093 (0.060)	-0.158*** (0.061)
CHAM hospitals	0.154a*** (0.060)	0.132a** (0.067)	0.024 (0.058)	-0.022 (0.090)	-0.108 (0.088)	-0.13 (0.083)
CHAM health centers	-0.071a (0.053)	0.069b (0.063)	0.091 (0.081)	0.140* (0.082)	0.022 (0.104)	0.162* (0.099)
Private health facilities	0.102a (0.113)	0.099b (0.100)	0.096 (0.112)	-0.003 (0.151)	-0.003 (0.151)	-0.006 (0.159)

Note : *CI* concentration index, *SE *standard errors; dominance test: a = dominance, b= non-dominance, c= concentration curve and line of equality cross; +: for private health facilities, only the OOP expenditure was included

*, **, *** statistically significant at the 10, 5, and 1% levels, respectively

Table 7 shows the change in the magnitude of the inequality of public and overall spending on institutional delivery across health care provider typology over time. Total public spending shifted to a low pro-poor inequality in 2015 from equality in 2004 and 2010. In contrast, total overall spending oscillated from equality in 2004 to a low-least poor inequality and shifted to equality again in 2015. Public hospitals remained slightly pro-poor at all years for both public and overall spending. Public spending at public health centers shifted from a low pro-poor inequality in 2004 and 2010 to moderate pro-poor inequality in 2015, whereas overall spending at public health centers remained at a low pro-poor inequality at all years. The inequality of overall spending at CHAM hospitals decreased continually from a moderate least poor inequality in 2004, though low-least poor inequality in 2010 to equality in 2015. Overall spending at CHAM health centers and private health facilities remained equal at all years.

**Table 7:** Changes of the inequality magnitude of public and overall spending on institutional delivery across health care provider typology

Health spending	Year	All health facilities	Public health facilities	Public hospitals	Public health centers	CHAM health facilities	CHAM hospitals	CHAM health centers	Private health facilities
Public spending	2004	Equal	Equal	Low least poor	Low pro-poor	n/a	n/a	n/a	n/a
	2010	Equal	Equal	Low least poor	Low pro-poor	n/a	n/a	n/a	n/a
	2015	Low pro-poor	Low pro-poor	Low least poor	Moderate pro-poor	n/a	n/a	n/a	n/a
Overall spending	2004	Equal	Equal	Low least poor	Low pro-poor	Low least poor	Moderate least poor	Equal	Equal
	2010	Low least poor	Equal	Low least poor	Low pro-poor	Equal	Low least poor	Equal	Equal
	2015	Equal	Low pro-poor	Low least poor	Low pro-poor	Equal	Equal	Equal	Equal

Notes: Non-significant CIs were considered equal, 1- -0.346: High pro-poor, -0.345 - 0.150: Moderate pro-poor, -0.149-0: Low pro-poor, 0.346-1: High least-poor, 0.150-0.345: Moderate least-poor, 0-0.149: Low least poor. n/a: not applicable

The seasonality analysis indicated no statistically significant changes to our analysis - meaning that the seasonal variations in health service utilization had no impact on the socioeconomic distribution of annualized curative services and institutional delivery included in our study.

## Discussion

Our study presents the results of a quasi-longitudinal analysis assessing the distributional incidence of both public and overall health spending on curative services and institutional delivery in Malawi at three periods. The study provides the first assessment of the distribution of health spending across socioeconomic groups at different types of health facilities in Malawi. Three key findings emerge from our analysis. First, we observe increased equality in the distribution of public and overall health spending over time for both curative services and institutional delivery. Second, the distributional incidence of public spending tended to be more egalitarian than overall spending throughout the study period. Third, both public and overall spending were more egalitarian for institutional delivery than for curative services, more egalitarian at public than at private health facilities, and more egalitarian at lower levels of care (e.g., health centers) than at the higher level of care (e.g., hospitals). Before we appraise our findings, it is important to note that the analytical approach we have used makes it impossible to attribute the observed patterns in distributional incidence to any one specific UHC reform in Malawi. We can only relate the distribution patterns observed over time to the different relevant health policies implemented in Malawi. Recognizing the inability of the BIA methodology to relate to specific policy actions and demand and supply behaviors [35], we are aware that additional analyses, including experimental behavioural models and political economy analyses [39], are needed to complement our work and shed further light of the role of the single health policies implemented in Malawi in fostering greater equality over time. Likewise, we also recognize that more detailed cost data would be needed to enable more detailed analyses, differentiating the distributional incidence by age groups.

We note that public spending was egalitarian and became increasingly so over time which is not aligned with findings from several prior studies conducted in other LMICs, where public spending has repeatedly been observed to benefit disproportionately the least poor for both curative services [2, 40–42] and institutional delivery [43]. This observation suggests that the free healthcare policy, making services available free of charge at point of use, strengthened by the institutionalization of the essential health package, is likely to have fostered equality in the distribution of public spending for both curative and institutional delivery services. The free care at public facilities and CHAM facilities through SLA contracts with the Malawi government has likely translated into the steady increase of the use of curative [26] and maternal [44, 45] services. Similar findings were reported by two studies conducted in India, which revealed that the introduction of the Janani Suralesha Yojana (JSY) policy enabling access to institutional delivery free of charge fostered equality in public health spending [46, 47]. Considering that the widespread reach of the free healthcare policy was achieved through direct contracting of CHAM facilities, our findings corroborate the already existing evidence on the importance of investing in policies that build on public-private partnerships. In contexts where the reach of public facilities is constrained, setting up such partnerships represents an essential step to ensure greater equality in both health care use and distribution of public health spending [48].

Nonetheless, we note that while becoming more egalitarian over time, the distribution of overall spending remained pro-least-poor for curative services but not for institutional delivery. This finding is consistent with findings from Ghana [49] and South Africa [50], but not from Tanzania [51], where overall health spending was also observed to be equally distributed. The persistent OOPE can probably explain this remaining inequality even in the presence of a formal free healthcare policy [27, 29]. The fact that inequality was observed mostly at non-public facilities is aligned with prior literature suggesting that OOPE in Malawi is driven mainly by people re-directing demand towards private services when human resources and medical supplies are absent at public facilities [22, 24]. The fact that this inequality is less dominant for institutional delivery is likely due to the introduction of SLA in 2006, enabling private not-for-profit facilities to provide maternal care free of charge. This fostered increased equal access to care for delivering women [44, 45] and possibly also to introducing the two PBF programs, both of which had a strong focus on maternal and reproductive health services [31]. Hence, our observation is likely due to the higher OOPE associated with using curative services compared to institutional delivery. In the current study, the share of OOPE incurred by the top quintile group for using curative services compared to the share of the bottom quintile group is approximately 67% higher than the share of OOPE incurred by making use of institutional delivery. People still incur some OOPE for curative health services in Malawi due to the persistently high prevalence of HIV and its concurrent infections, the emergence of high-cost treatment for non-communicable diseases, and the introduction of fees in private wards [26] in response to chronic underfunding of the EHP [22, 23, 27, 52]. Therefore, our findings call for removing instead of introducing financial barriers at the point of care, especially for the poorer segments of the population [53]. Also, the increasing public and donor funding should be channelled towards sustaining the implementation of the EHP and expanding SLAs to include services other than maternal care as the only means to increase accessibility to free care for curative services.

Being a low-income country, Malawi faces challenges to finance all needed human and material resources and expand the provision of free care to all health services to address existing inequalities in the distribution of health benefits. At the moment, per capita spending on health remains low at US$39, which is equivalent to approximately 8% of the domestic budget [15, 17]. Moreover, development partners contribute approximately 60% of the health budget [54]. There is need to increase public health spending substantially to meet at least the level postulated by the Abuja Declaration, set at 15% of a country national budget [16]. In an emergency situation, such as the one induced by the current COVID-19 pandemic, the underfunding of the Malawi health system is likely to result in even greater inequalities than the ones detected by our study. Since our analysis precedes the pandemic, further studies are needed to examine how the pandemic might have affected the distribution of health benefits in the country.

Confirming previous results from low-and-middle-income countries [55–57], we identified a noticeably higher inequality at higher levels of care (e.g., hospital) for both curative services and institutional delivery. Across SSA, higher-level health facilities are concentrated in urban areas, often only accessible to the least poor, given the higher direct and indirect costs of seeking care at this level [58], including considerable transport costs [59–61]. For instance, a study by Nakovics and colleagues [27] indicated that transport costs in Malawi represent as much as 43% of the total cost of care. Our findings indicate the need for action on the supply-side by increasing the density of secondary level facilities and on the demand-side by introducing reimbursements for transport costs to overcome existing inequalities due to geographical disparities [59].

### Methodological considerations

Despite its value as the first study to explore the distributional incidence of health spending in Malawi, we recognize that our study has some limitations. First, DHS and IHLCS household surveys contain different information to allow for the classification of individuals across socioeconomic groups. We used consumption expenditure and material asset ownership to classify the individuals in socioeconomic quintiles for IHLCS and DHS, respectively. The resulting socioeconomic groups may not be fully comparable across IHLC and DHS surveys. However, we assume that any potential difference may be insignificant since prior research has indicated how in low-income countries like Malawi, the magnitude of household consumption expenditures mirrors households' ownership of material assets [62, 63]. Second, based on the data at our disposal, having applied the constant unit subsidy assumption, we might have masked differences in financial health benefits accruing to people of different socioeconomic status or living in different geographical settings. Third and last, our study does not account for differential health care needs across socioeconomic groups (horizontal equity) nor differences in the age, gender and quality of the services received. For more decomposition of the socioeconomic inequality in health spending in Malawi, further analysis relying on comprehensive data including health care needs, age, gender and the quality of health services is needed.

## Conclusion

Though the inequality in health spending on curative services and institutional delivery in Malawi has decreased over time, our study depicts that disparities in the distribution of public and overall health spending persist. Malawi's critical challenge is that of reducing or eliminating the out-of-pocket payments that still hinder poorer segments of the population from using health services. The establishment of an EHP ensuring the provision of essential services free of charge at the point of use represents a first critical step in ensuring access to care across all population groups. However, insufficient funding may hamper its effective implementation [15, 17]. Hence, greater investments enabling effective implementation are needed to tackle persisting inequalities in healthcare access to foster greater equality in the distribution of health benefits.

## Supplementary Information

**Additional file 1.**

## Data Availability

The original datasets from DHS (http://dhsprogram.com/) and IHLCS (https://microdata.worldbank.org/) are freely available. The original datasets from NHA are available from the corresponding author upon reasonable request and with permission of Malawi's Ministry of Health.

## Abbreviations

BIA: Benefit Incidence Analysis
CHAM: Christian Health Association of Malawi
CI: Concentration Index
DHS: Demographic and Health Surveys
EHP: Essential Health Package
IHLCS: Integrated Household Living Condition Surveys
NHA: National Health Accounts
OOPE: Out-Of-Pocket Expenditure
RBF4MNH: Results-Based Financing for Maternal and Newborn Health
SLA: Service-Level Agreements
SSA: Sub-Saharan Africa
SSDI-PBI: Support for Service Delivery Integration Performance-Based Incentive
UHC: Universal Health Coverage
USD: United States Dollar
