# Supplementary material for: How equitable is health spending on curative services and institutional delivery in Malawi? Evidence from a quasi-longitudinal benefit incidence analysis
Source: Int J Equity Health. 2022 Feb 18;21:25. doi: 10.1186/s12939-022-01624-5 (PMC8856874; doi:10.1186/s12939-022-01624-5)
Supplement: Supplementary file 1 — Additional file 1. [file 12939_2022_1624_MOESM1_ESM.docx]

**Additional file 1**

**Table S1: Summary information on population survey data employed in the study**

| **Health service utilization indicator** | **Socioeconomic status indictor** | **Household survey** | **Year** | **When the survey was conducted** | **Sampling strategies** |
| --- | --- | --- | --- | --- | --- |
| Use of curative services by level of care and stratified by socio-economic status | Household consumption expenditure on food and non-foods items | Integrated Household Living Condition surveys (IHLCS) | 2004 | March 2004 - March 2005 | Stratified two-stage sampling technique:  In the first stage, the primary units or enumeration areas (EAs) were drawn to probability proportional to the number of households counted in the EA (for a total of approximately 779 EAs).  In the second stage, households were drawn in equal probability in each of the enumeration areas (for a total of approximately 12,480 households). |
|  |  |  | 2010 | March 2010 - March 2011 |  |
|  |  |  | 2016 | April 2016 -April 2017 |  |
| Use of institutional delivery by level of care and stratified by socio-economic status | Household ownership of material assets | Demographic and Health Survey (DHS) | 2004 | October 2004 -January 2005 | Stratified two-stage sampling technique:  In the first stage, 850 EAs were randomly selected with probability proportional to the SEA size. An EA is a convenient geographical area with an average size of 235 households.  In the second stage, households were drawn with equal probability in each of the enumeration areas (for a total of approximately 27,518 households). |
|  |  |  | 2010 | June - November 2010 |  |
|  |  |  | 2015 | October 215 -January 2016 |  |

**Table S2**. Percentage share of annual utilization of curative health services by quintiles and facility type (2004, 2010 and 2016)

| Socioeconomic quintile | **2004** (n =5 0,882) | | | **2010** (n = 19,868) | | | **2016** (n = 53,873) | | |
| --- | --- | --- | --- | --- | --- | --- | --- | --- | --- |
|  | Public health facilities | CHAM health facilities | Private health facilities | Public health facilities | CHAM health facilities | Private health facilities | Public health facilities | CHAM health facilities | Private health facilities |
| 1 (Poorest) | 16.84 | 10.48 | 10.38 | 17.48 | 14.07 | 11.12 | 19.47 | 6.23 | 7.18 |
| 2 | 21.54 | 15.58 | 9.48 | 16.53 | 15.56 | 10.16 | 19.47 | 18.16 | 11.04 |
| 3 | 20.55 | 20.25 | 20.96 | 21.34 | 14.81 | 20.45 | 21.16 | 22.49 | 15.32 |
| 4 | 21.75 | 25.99 | 20.47 | 21.58 | 17.78 | 20.16 | 22.16 | 20.87 | 24.22 |
| 5 (Least poor) | 19.32 | 27.70 | 38.71 | 23.07 | 37.78 | 38.10 | 17.73 | 32.25 | 42.24 |

**Table S3a**. Percentage share of annual utilization of institutional delivery service by quintiles and facility type (2004 and 2010)

| Socioeconomic quintile | **2004** (n = 35,883) | | | | | **2010** (n = 72,286) | | | | |
| --- | --- | --- | --- | --- | --- | --- | --- | --- | --- | --- |
|  | Public hospitals | Public health centers | CHAM health centers | CHAM hospitals | Private health facilities | Public hospitals | Public health centers | CHAM health centers | CHAM hospitals | Private health facilities |
| 1 (Poorest) | 16.72 | 22.08 | 20.81 | 10.88 | 14.21 | 13.40 | 23.81 | 18.32 | 17.84 | 8.02 |
| 2 | 15.95 | 21.13 | 23.00 | 16.87 | 9.97 | 18.57 | 21.08 | 21.02 | 18.73 | 16.95 |
| 3 | 16.74 | 21.18 | 24.30 | 17.29 | 18.07 | 20.65 | 22.37 | 24.32 | 15.14 | 25.65 |
| 4 | 17.47 | 18.79 | 20.34 | 19.99 | 9.75 | 20.87 | 15.49 | 20.25 | 17.88 | 16.17 |
| 5 (Least poor) | 33.13 | 16.82 | 11.54 | 34.97 | 48.00 | 26.51 | 17.24 | 16.08 | 30.42 | 33.21 |

**Table S3b**. Percentage share of annual utilization of institutional delivery service by quintiles and facility type (2015)

| Socioeconomic quintile | **2015** (n = 68,074) | | | | |
| --- | --- | --- | --- | --- | --- |
|  | Public hospitals | Public health centers | CHAM health centers | CHAM hospitals | Private health facilities |
| 1 (Poorest) | 17.61 | 26.70 | 26.20 | 17.54 | 8,97 |
| 2 | 20.21 | 25.64 | 27.60 | 23.35 | 17.55 |
| 3 | 16.78 | 19.27 | 19.70 | 16.60 | 9.52 |
| 4 | 18.78 | 16.19 | 16.04 | 19.64 | 25.98 |
| 5 (Least poor) | 26.67 | 12.20 | 10.46 | 22.87 | 37.98 |
